# Higher arterial pressure during cardiopulmonary bypass may not reduce the risk of acute kidney injury

**DOI:** 10.1186/s13019-019-0929-4

**Published:** 2019-06-13

**Authors:** Kristian Kandler, Jens C. Nilsson, Peter Oturai, Mathias E. Jensen, Christian H. Møller, Jens Otto Clemmesen, Henrik C. Arendrup, Daniel A. Steinbrüchel

**Affiliations:** 1 Department of Cardiothoracic Surgery, Copenhagen University Hospital, Rigshospitalet, Blegdamsvej 9, 2100 Copenhagen, Denmark; 2 Department of Cardiothoracic Anaesthesiology, Copenhagen University Hospital, Rigshospitalet, Copenhagen, Denmark; 3 Department of Clinical Physiology, Nuclear Medicine and PET, Copenhagen University Hospital, Rigshospitalet, Copenhagen, Denmark; 4 Department of Hepatology, Copenhagen University Hospital, Rigshospitalet, Copenhagen, Denmark

**Keywords:** Acute kidney injury, Arterial pressure, Cardiac surgery

## Abstract

**Background:**

Acute kidney injury after cardiac surgery is common and associated with increased mortality. It is unknown whether an intended higher arterial pressure during cardiopulmonary bypass reduces the incidence of acute and chronic kidney injury.

**Methods:**

Patients were randomised either to a control group or a high pressure group (arterial pressure > 60 mmHg). The inclusion criteria were age > 70 years, combined cardiac surgery and serum creatinine < 200 μmol/L. Glomerular filtration rate using the Cr-EDTA clearance method was measured the day before surgery and 4 months postoperatively. The RIFLE criteria were used to define the presence of acute kidney injury. In addition, the ratio between urinary Neutrophil Gelatinase-Associated Lipocalin (NGAL) and creatinine was measured.

**Results:**

Ninety patients were included. Mean age was 76 ± 4 years and 76% were male. Mean arterial pressure was 47 ± 5 mmHg in the control group and 61 ± 4 mmHg in the high pressure group (*p* < 0.0001). The change in glomerular filtration rate at follow-up was − 9 ± 12 ml/min in the control group and − 5 ± 16 ml/min in the high pressure group (*p* = 0.288, 95% CI − 13 to 4). According to the RIFLE criteria 38% in the control group and 46% in the high pressure group developed acute kidney injury (*p* = 0.447). The postoperative urinary NGAL/creatinine ratio was comparable between the groups.

**Conclusions:**

An intended increase in arterial pressure during cardiopulmonary bypass to > 60 mmHg did not decrease the incidence of acute or chronic kidney injury after cardiac surgery.

**Trial registration:**

Clinicaltrials.gov, identifier: NCT01408420. Registered 3rd of August 2011.

## Background

Acute kidney injury (AKI) is a well-known complication to cardiac surgery and is associated with increased mortality [1–6]. The reported incidences range from 1 to 56% in the literature [7–9].

The etiology behind the development of AKI after cardiac surgery is incompletely understood and probably multifactorial. Possible contributing factors include systemic inflammatory response, microemboli and haemodilution. Some studies have also indicated that the use of a heart-lung machine versus off-pump beating heart surgery increases the risk of AKI [10–12].

Furthermore, the management of flow and arterial pressure (AP) during cardiopulmonary bypass (CPB) is not evidence based [13]. It has been shown in a previous study that a prolonged duration with an AP < 60 mmHg during CPB increases the risk of AKI [14]. The range of renal autoregulation has been found to be between 75 mmHg and 160 mmHg in an experimental setting [15]. During CPB the AP is below this range the majority of the time. Furthermore, the pressure during CPB is continuous and not pulsatile as under normal conditions and the pressure spikes of the systolic blood pressure are not present. Therefore, when outside of the autoregulatory range of the kidneys, a higher-than-spontaneous AP during CPB may improve renal microcirculation and decrease the risk of AKI. In addition, it is unknown whether there is a correlation between AKI and chronic kidney injury after cardiac surgery.

The hypothesis of this study is that an intended high AP during CPB decreases the incidence of acute and chronic kidney injury in a population highly predisposed to AKI.

## Methods

Ninety patients were enrolled in the study between May 2011 and December 2013 (clinicaltrials.gov identifier: NCT01408420). All patients were operated at Rigshospitalet, Copenhagen University Hospital, Copenhagen, Denmark. The study was approved by The Regional Committee on Biomedical Research Ethics. Written informed consent was received from all patients prior to their inclusion in the study.

### In- and exclusion criteria

To ensure that the cohort had a high preoperative risk of developing AKI, the inclusion criteria were age > 70 years and complex cardiac surgery procedures (heart valve and bypass surgery in combination). Exclusion criteria were serum creatinine (sCr) > 200 μmol/L, previous heart surgery, endocarditis and acute operation defined as coronary angiography < 24 h of surgery.

### Randomisation

A parallel-group study was conducted where patients were randomised into either a control group (CG) or a high pressure group (HPG) using closed opaque envelopes that were sequentially numbered. The allocation ratio was 1:1. Randomisation was performed the day before surgery after informed consent. The patients and outcome assessors who analysed GFR, blood- and urine samples were blinded until follow-up. The generation of the random allocation sequence, the randomisation and enrolment of patients were done by the first author of this paper.

### Anaesthesia

Patients were premedicated with triazolam 0.125–0.250 mg orally 1 hour prior to surgery. Anaesthesia was induced with fentanyl (10 μg/kg), propofol (1–2 mg/kg) and cisatracurium (0.1 mg/kg). Maintenance of anaesthesia was achieved using sevoflurane (0.5–3%) and continuous infusion of remifentanil (15 to 30 μg/kg/hour). Intravenous administration of 1500 mg of cefuroxime and a single-shot of 240 mg of gentamicin were given after induction of anaesthesia. No specific guidelines were used in regards to the administration of the single-shot gentamicin. AP was recorded through a cannula placed in the radial artery.

After heparinisation (350 IU/kg, ACT > 480 s), normothermic CPB (36.5–37.0 °C bladder temperature) was initiated as follows: in the ascending aorta an angled arterial cannula was placed (DLP 24 FR, Medtronic, Minneapolis, Minnesota) and a two-stage venous cannula (36/46 FR, Medtronic) was inserted through the right atrial appendage. A membrane oxygenator (Capiox RX25, Terumo, Tokyo, Japan) and a roller pump (Stockert S5, Sorin Group, Milano, Italy) were used for perfusion with non-pulsatile flow. The arterial line included a 40-μm filter (AL06, Pall, Port Washington, New York). Pump flow was calculated by multiplying 2.4 L/minute by the body surface area in square metres.

Intraoperative CPB data were retrieved from electronic perfusion charts. The AP during CPB was sampled electronically every minute by the heart-lung machine. The average AP during CPB was calculated using the values from clamping to de-clamping of the ascending aorta.

### Endpoints

The primary end-point of this study was mean change in glomerular filtration rate (GFR) at follow-up compared to baseline.

The secondary end-point were change in urinary Neutrophil Gelatinase-Associated Lipocalin (uNGAL) and urinary creatinine ratios at different postoperative time-points compared to baseline, a method that has been shown to be highly sensitive in predicting AKI [16].

It was not possible to calculate sample size since no data on ^51^Cr-ethylenendiaminetetra acetic acid plasma clearance technique (Cr-EDTA) GFR measurements existed on cardiac surgery patients at the initiation of the study.

Baseline u-NGAL and urine creatinine were measured using urine samples taken just after induction of anaesthesia and insertion of a urinary catheter. Postoperatively the samples were taken at arrival to the intensive care unit (ICU) and 6-, 18-, 48- and 120 h postoperative. The samples were centrifuged for 5 min at 1000 RPM and the supernatant was pipetted into cryotubes. The cryotubes were placed in a freezer at − 80 degrees Celsius for a maximum of 9 months before analysis, a period that has previously been found to be safe for storing uNGAL [17]. The urine samples were analysed for uNGAL using the NGAL Test reagent kit (BioPorto Diagnostics, Gentofte, Denmark) on a Cobas c501 analyser (Roche, Basel, Switzerland) using fully automated particle-enhanced turbidimetric immunoassay. On the same analyser urine creatinine levels were measured to adjust for postoperative hydration status using the Creatinine Plus version 2 reagent kit (CREP 2, Roche, Basel, Switzerland).

GFR was measured by the ^51^Cr-ethylenendiaminetetra acetic acid (EDTA) plasma clearance technique the day before surgery and 4 months postoperative [18]. Four plasma samples were collected during 60 min within the period of three to 5 hours after tracer injection.

Baseline sCr values were defined as the preoperative value closest to the day of surgery. Postoperative sCr samples were taken in the morning of the first and second postoperative day. The patients were characterised as either AKI or no-AKI based on the RIFLE criteria. AKI was present when an increase in sCr values of > 50% or absolute increase of > 27 μmol/l occurred within the first 48 h postoperative, compared to baseline. An estimated GFR (eGFR) was calculated based on sCr using the Cockroft-Gault formula. In accordance with the RIFLE criteria an eGFR decrease of > 25% was also used to define AKI.

### Intervention

In the CG the patients underwent standard anaesthesia and CPB with the exception of a maximum of 110% flow on the heart-lung machine.

In the HPG an infusion containing isotonic sodium chloride and norepinephrine was mixed according to patient weight so that 1 ml/hour equalled 0.01 μg/kg/min of norepinephrine. This mixture was used when the AP was < 60 mmHg during CPB. If the target pressure could not be reached the infusion rate was not increased above 30 ml/min. Maximum flow on the heart-lung machine was 110%.

### Statistics

The study was conducted on an intention-to-treat basis.

Continuous data are presented as means ± standard deviations (SD) or median (interquartile range). Continuous variables were compared by Student’s unpaired *t*-test; categorical variables were compared by Pearson’s chi-squared test. Mann-Whitney U-test was used to compare sCr levels between the groups and u-NGAL/creatinine ratios between the groups at each time-point after correcting for the preoperative measurement. Paired samples *t*-test was used to compare preoperative GFR, eGFR and sCr with values at follow-up.

Differences were considered to be statistically significant when the *p* value was < 0.05.

Statistical analysis was performed using the statistical software package SPSS, version 22.0.0.0; SPSS Inc.; Chicago IL.

## Results

Ninety patients were included from May 2011 to October 2013. Last patient follow-up was done January 2014. Mean age was 76 ± 4 years and 76% were male.

No differences in preoperative data were found between the groups (Table 1). Intraoperative data were comparable between the groups except the AP, which was 47 ± 5 mmHg and 61 ± 4 mmHg in the CG and HPG, respectively (*p* < 0.001) (Table 2). Postoperative data, including short- and medium term survival, incidence of acute kidney injury and GFR were equal between the groups (Table 3).

**Table 1:** Preoperative data

	CG	HPG	*p* value	95% CI
N	45	45	–	
Male gender	33 (73%)	32 (71%)	0.450	
Age (years)	76.3 ± 4.2	76.6 ± 4.7	0.731	−2 to 2
BSA (m^2^)	1.93 ± 0.19	1.91 ± 0.18	0.611	− 0.07 to 0.10
BMI (kg/m^2^)	26.5 ± 3.70	27.2 ± 4.70	0.519	−2.5 to 1.1
EuroSCORE II	3 (2–5)	3 (3–5)	0.264	−1 to 1
Hypertension	31 (80%)	26 (68%)	0.268	
COPD	2 (5%)	0 (0%)	0.157	
NIDDM	3 (8%)	8 (21%)	0.094	
IDDM	2 (5%)	3 (8%)	0.622	
PAD	7 (18%)	3 (8%)	0.189	
CD	6 (15%)	5 (13%)	0.780	
GFR (ml/min)	77 ± 21	69 ± 28	0.142	−3 to 19
sCr (μmol/dl)	88 (80–101)	84 (69–116)	0.452	−18 to 12
eGFR (ml/min)	72 ± 22	71 ± 29	0.966	− 11 to 11

Percentages are given as total within group. Continuous data are presented as mean ± SD or median (interquartile min – max)

*BSA* Body surface area, *BMI* Body mass index, *CI* confidence interval, *COPD* Chronic Obstructive Pulmonary Disease, *IDDM* Insulin-Dependent Diabetes Mellitus, *NIDDM* Non-Insulin-Dependent Diabetes Mellitus, *PAD* Peripheral Arterial Disease, *CD* Cerebral disease, *eGFR* Estimated glomerular filtration rate (calculated using the Cockroft-Gault formula), *GFR* Glomerular filtration rate (estimated by Cr-EDTA clearance), *sCr* Serum creatinine

**Table 2:** Intraoperative data

	CG	HPG	*p* value	95% CI
AP (mmHg)	47 ± 5	61 ± 4	< 0.001	−15 to − 11
Flow (L/min/m2)	2.6 ± 0.2	2.6 ± 0.2	0.810	− 0.1 to 0.1
CABG + AVR	38 (84%)	36 (80%)	0.415	
CABG + MVR	4 (9%)	8 (18%)	0.290	
AVR + MVR	1 (2%)	0 (0%)	0.314	
CPB time (min)	130 ± 36	130 ± 31	0.994	−30 to 19
XC time (min)	100 ± 39	93 ± 22	0.310	−19 to 12
Blood loss (ml)	636 ± 482	667 ± 397	0.759	− 222 to 153
Diuresis (ml)	517 ± 294	639 ± 438	0.152	− 299 to 15
Change in HCT (%)	−8 ± 5	−7 ± 4	0.069	−4 to 0
Fluid balance (ml)	1490 ± 1206	1246 ± 968	0.327	− 382 to 753
Gentamicin administered	31 (78%)	32 (80%)	0.353	

Percentages are given as total within group. Continuous data are presented as mean ± SD

*AP* Arterial pressure, *AVR* Aortiv valve replacement, *CI* confidence interval, *CPB* Cardiopulmonary bypass, *HCT* Haematocrit, *MVR* Mitral valve replacement, *XC* Aortic cross clamp

**Table 3:** Postoperative data

	CG	HPG	*p* value	95% CI
Delta sCr (mmol/L)	20 (6–51)	25 (10–49)	0.560	−18 to 18
Delta eGFR (ml/min)	−6 (− 15–4)	− 3 (− 13–5)	0.522	−7 to 5
AKI	16 (38%)	19 (46%)	0.447	
Dialysis	4 (10%)	3 (7%)	0.565	
Re-operation	4 (10%)	1 (3%)	0.165	
Stroke	3 (8%)	2 (5%)	0.687	
4 months follow-up				
Change in GFR (ml/min)	− 9 ± 12	−5 ± 16	0.288	−13 to 4
> 10% decrease in GFR^a^	11 (44%)	9 (39%)	0.732	
30-day mortality	3 (7%)	3 (7%)	1.000	
6-months mortality	7 (16%)	8 (18%)	0.763	

Percentages are given as total within group. Continuous data are presented as mean ± SD or median (interquartile min – max)

*AKI* Acute kidney injury, *CI* confidence interval, *AP* Arterial pressure, *eGFR* Estimated glomerular filtration rate (calculated using the Cockroft-Gault formula), *GFR* Glomerular filtration rate (estimated by Cr-EDTA clearance), *sCr* Serum creatinine

^a^Percentages of total of patients at follow-up

Forty-eight patients completed follow-up (25 patients in the CG and 23 in the HPG). Six (7%) patients died in-hospital (three patients in each group). Nine patients (10%) died after discharge but before follow-up (4 patients in the CG group and 5 patients in the HGP group). Four (4%) patients declined to continue in the trial in the immediate postoperative period (two in each group). The actual follow-up rate was therefore 48 out of 71 patients (68%). Time of follow-up was 4.1 ± 1.5 months.

**Fig. 1:**
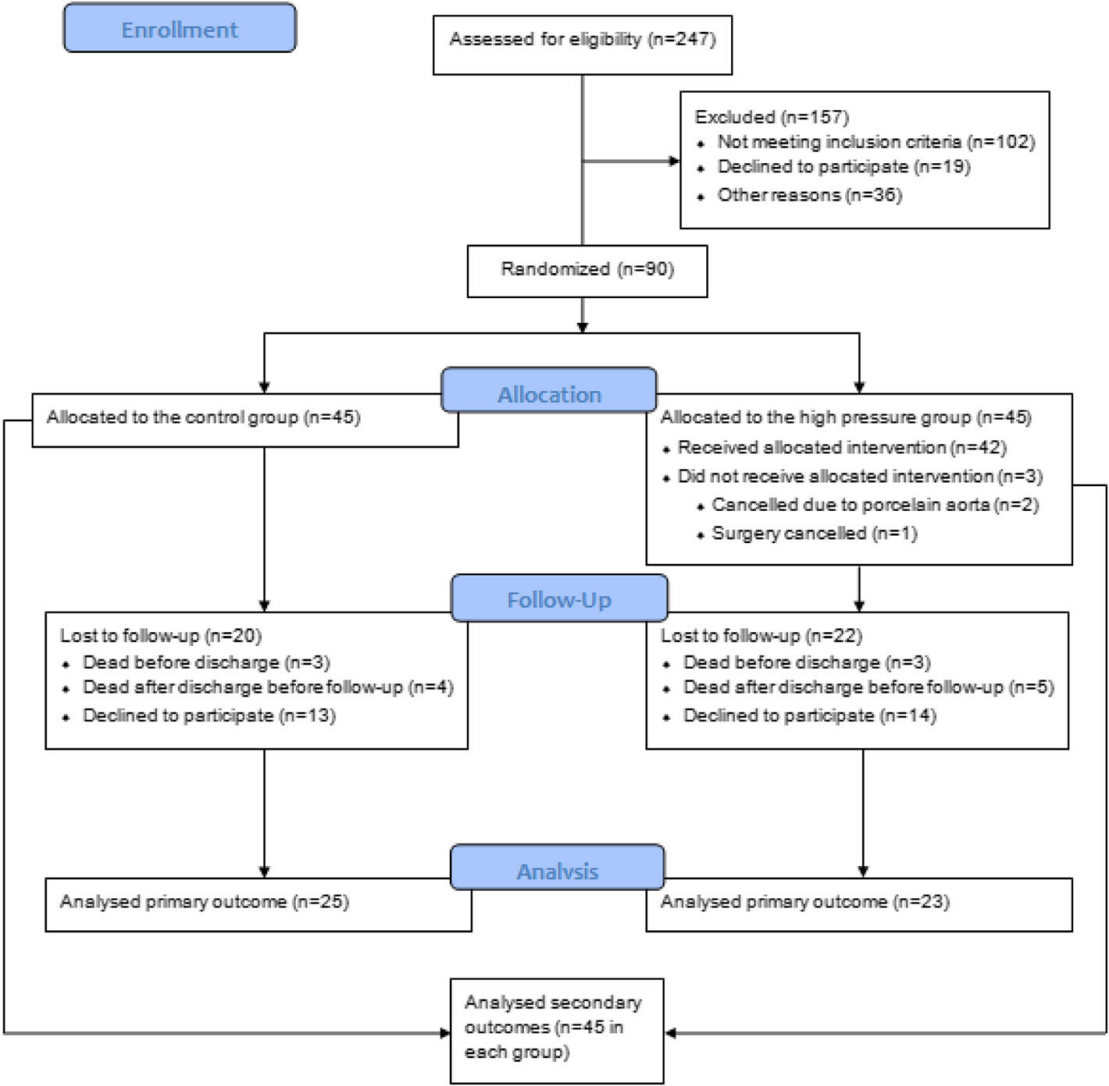
Participant flow diagram

The secondary outcomes were analysed in all 90 patients. A participant flow diagram is presented in Fig. 1.

### Glomerular filtration rate

For the total patient group a significant decline in GFR following cardiac surgery was observed.

Preoperative and postoperative GFR was 78 ± 27 ml/min and 70 ± 26 ml/min, respectively (*p* = 0.001). The GFR change from baseline to follow-up did not differ between the two groups, − 9 ± 12 ml/min in the CG and − 5 ± 16 ml/min in the HPG (*p* = 0.288) (Table 3).

Eleven patients (44%) in the CG and 9 (39%) in the HPG had a GFR drop of > 10% at follow-up compared to baseline (*p* = 0.732). At follow-up 9 patients (36%) in the CG group and 5 (22%) in the HPG had a GFR drop of > 20% (*p* = 0.278).

### Acute kidney injury

The uNGAL/creatinine ratio was not significant between the two groups at any time-point (Table 4).

**Table 4:** uNGAL/creatinine changes from baseline (ng/mL)

	CG	HPG	*p* value
Preoperative (baseline)	2 (1–4)	2 (1–5)	0.824
Postoperative	42 (11–141)	40 (19–141)	0.720
6 h	8 (4–58)	10 (2–40)	0.768
18 h	1 (0–6)	2 (0–8)	0.744
48 h	3 (0–6)	4 (1–6)	0.596
120 h	2 (0–5)	4 (1–10)	0.301

Data is presented as median (interquartile min – max)

35 (41%) of the patients developed AKI according to the RIFLE criteria. No significant differences were found in the incidence of AKI between the groups which was 16 (38%) and 19 (46%) in the CG and HPG respectively (*p* = 0.447). 7 patients received renal replacement therapy during the hospital stay of which 4 (9%) were in the CG and 3 (7%) in the HPG (*p* = 0.565)

Among AKI patients, 12 out of 17 (71%) had a decrease in GFR > 10% at follow-up. In patients who did not develop AKI 8 out of 31 (26%) patients had a GFR decrease > 10%. The difference in proportion of patients with a GFR decrease > 10% were statistically significant between AKI and no-AKI patients (*p* = 0.003).

The results of a sub-analysis comparing patients who develop AKI with patients who do not develop AKI looking at specific risk factors are presented in Table 5. No significant risk factors of AKI were identified.

**Table 5:** Subanalysis of potential risk factors of AKI

	No-AKI	AKI	*p* value
N	51	36	
Male gender	35 (69%)	29 (80%)	0.176
Age	76 ± 4	77 ± 5	0.098
BMI (kg/m^2^)	26 ± 4	28 ± 5	0.061
EuroSCORE II	3 (2–5)	3 (2–4)	0.865
GFR	73 ± 28	74 ± 21	0.867
sCr	87 (75–104)	85 (75–109)	0.836
eGFR	67 ± 28	75 ± 24	0.171
AP	54 ± 8	54 ± 8	0.802
CPB time	124 ± 29	138 ± 37	0.073
Gentamicin administered	38 (83%)	24 (73%)	0.155

Percentages are given as total within AKI-group. Continuous data are presented as mean ± SD or median (interquartile min – max)

*AKI* Acute kidney injury, *AP* Arterial pressure, *BMI* Body mass index, *CPB* Cardiopulmonary bypass, *GFR* Glomerular filtration rate, *HCT* Haematocrit, *XC* Aortic cross clamp

Cause of death after discharge but before follow-up was cardiac related death in three patients and one patient in the CG and HPG, respectively. One patient in each group died from infection. One patient in the CG died from intracerebral haemorrhage. Dehydration and cancer was the cause of death in two patients in the HPG. No patients that died before follow-up were in the need of dialysis. No significant difference in the cause of death was found between the groups. Data on mortality are shown in Table 3.

## Discussion

We present a study conducted on cardiac surgery patients who had a high risk of developing AKI by using age > 70 years and complex cardiac procedures as inclusion criteria. To our knowledge this is the first randomised study on the subject that have long-term renal outcome as the primary outcome, not only by looking at sCr or eGFR, but using measured GFR by using the Cr-EDTA clearance method.

### Long-term renal function

A significant loss of GFR in both groups was observed at follow-up. Cardiac surgery should therefore be considered not only a risk factor for developing AKI but also a risk factor of permanent loss of renal function.

The primary finding of this study was that an intended increase in AP to > 60 mmHg during CPB using norepinephrine did not result in any significant differences in GFR change from preoperative values to follow-up compared to lower intraoperative arterial pressures. This suggests that AP during CPB does not influence long-term renal function. However, overall measured GFR had decreased nearly 10% on average 4 months after surgery. Additionally, the development of AKI < 48 h postoperative was associated with a GFR reduction of > 10% at follow-up. Cardiac surgery should therefore be considered not only a risk factor for developing AKI but seems to result in a permanent loss of renal function in elderly patients undergoing complex cardiac surgery. This permanent loss of renal function has not been previously described and should be taken into consideration when planning surgery in this group of patients.

### Short-term renal function

The secondary objective of this study was to investigate the relationship between AP during CPB and short-term renal function, defined by uNGAL/creatinine ratio and AKI.

By using inclusion criteria defining individuals in high risk of AKI, the study population had an AKI incidence of 41% in total. The reported incidence of AKI in the literature is 1–56% [7–9].

The incidence of AKI, estimated by the RIFLE criteria, was found to be equal in the two groups. The ratio of uNGAL/creatinine was also equal between the groups at all time-points. The uNGAL/creatinine ratio was significantly increased immediately postoperative and 6 h postoperative, indicating damage to the renal parenchyma due to intraoperative factors.

Only a few randomised studies exist investigating arterial pressure during CPB in relation to kidney injury as a primary outcome. One study used no age restrictions and included both combined and non-combined valve and CABG surgery [19]. Patients were randomised into three AP groups, ranging from an AP < 60 mmHg to > 70 mmHg. The primary endpoint was AKI defined by the RIFLE criteria. Eleven percent of the patients developed AKI. No differences regarding the incidence of AKI were found between the three groups. Another study used age > 70 years as inclusion criterion as in our study, but only included CABG patients [20]. Patients were randomised into the same AP groups as in the above mentioned study. The incidence of AKI was equal between the groups. Both studies used the same lower AP threshold as in our study, i.e. < 60 mmHg.

A large randomized study conducted on 300 patients aimed for an even higher AP of 75–85 mmHg in the control group [21]. The target AP in the control group was 50–60 mmHg which is similar to the present study and the above mentioned studies. The outcome was AKI defined by changes in sCr. The rate of AKI in the groups was 17% vs. 17% (*p* = 1). The mean AP reached in the control group was 60 ± 6 mmHg, which is close to the AP in the control group of our study. This supports our findings and suggests that even very high AP during CPB does not protect the kidneys. At least not in terms of acute kidney injury.

A recent study with a more direct approach of measuring renal function by renal vein catheterization suggests that a higher flow than 2.4 L/min/m^2^ is beneficial for the kidneys [22]. The increased flow rates used were 2.7 L/min/m^2^ and 3.0 L/min/m^2^ which improved the renal oxygen supply/demand relationship by 14 and 30% respectively.

The above mentioned studies, in addition to our study, indicate that AP during CPB has no influence on the incidence of AKI on a broad range of patients and procedures. In addition, future randomised studies should also investigate the relationship between arterial flow and renal function.

### Limitations

This study used an AP of > 60 mmHg as the aim in the HPG. Therefore, it still remains unclear if even higher APs could decrease the risk of kidney injury.

The RIFLE criteria used to define AKI is based on sCr, which varies depending on age, gender, muscle mass and race. This is partly adjusted for since the patient’s own sCr is used as baseline and adjusted for age, gender and weight to calculate eGFR. However, in the postoperative period sCr can also vary considerably depending on the degree of haemodilution, making the values less reliable.

The number of patients lost to follow-up was high and the cohort size was relatively small. Although the numbers were the same in both groups there is still a risk of loss to follow-up bias.

It was not possible to perform a sample size calculation prior to the study since no previous data was available on GFR measurements on the patient category that was included in the study.

## Conclusion

An intended AP > 60 mmHg during CPB did not seem to decrease the risk of acute or chronic kidney injury in patients > 70 years of age undergoing concomitant cardiac surgical procedures. Cardiac surgery generally resulted in a substantial loss of renal function measured at follow-up 4 months postoperatively.

## Data Availability

The datasets used and analysed during the current study are available from the corresponding author on reasonable request.

## Abbreviations

AKI: Acute kidney injury
AP: Arterial pressure
CG: Control group
CPB: Cardiopulmonary bypass
GFR: Glomerular filtration rate
HPG: High pressure group
ICU: Intensive care unit
NGAL: Neutrophil Gelatinase-Associated Lipocalin
sCr: Serum creatinine
